# The influence of fatigue on work capacity and quality of life among miners with silicosis

**DOI:** 10.47626/1679-4435-2021-883

**Published:** 2023-08-08

**Authors:** Tamires Patrícia Souza, Inês Monteiro

**Affiliations:** 1 Faculdade de Enfermagem, Universidade Estadual de Campinas, Campinas, SP, Brazil; 2 Faculdade de Ciências da Saúde, Universidade Metodista de Piracicaba, Piracicaba, SP, Brazil

**Keywords:** silicosis, quality of life, fatigue, miners, silicose, qualidade de vida, fadiga, trabalhadores de mineração

## Abstract

**Introduction:**

Factors associated with the mining environment can contribute to work accidents and reduced caution at work, which may affect the quality of life and work capacity of miners.

**Objectives:**

To evaluate if fatigue influences quality of life and work capacity in miners with silicosis.

**Methods:**

This was a cross-sectional study conducted in the Northern Region of the state of Rio Grande do Sul, Brazil. Sociodemographic data and data on fatigue, work capacity, and quality of life were collected during the second half of 2017 and first half of 2018.

**Results:**

All participants were men. Mean participant age was 52.6 (± 7.2) years, and most (70.4%) of them did not finish elementary school. The strongest correlations were found between the physical, overall, and total fatigue domains and the World Health Organization Quality of Life – BREF and between the total and mental fatigue domains and the Work Ability Index. Strong correlations were also found between overall and total fatigue and the total St. George’s Respiratory Questionnaire score.

**Conclusions:**

Silicosis and physical workload have a negative impact on respiratory quality of life and perception of fatigue among miners.

## Abstract (pt)

**Introdução:**

Fatores associados ao ambiente de mineração podem contribuir para acidentes e menor autocuidado no trabalho, podendo afetar a qualidade de vida e a capacidade para o trabalho.

**Objetivos:**

Avaliar se a fadiga influencia a qualidade de vida e a capacidade para o trabalho dos trabalhadores de mineração com silicose.

**Métodos:**

Trata-se de estudo transversal, desenvolvido no norte do Rio Grande do Sul, Brasil. Foram coletados dados so cio demográficos, de fadiga, da capacidade para o trabalho e de qualidade de vida. A coleta de dados ocorreu durante o segundo semestre de 2017 e o primeiro semestre de 2018.

**Resultados:**

Todos os participantes eram homens. A idade média dos participantes foi de 52,6 (± 7,2) anos, e a maioria (70,4%) não concluiu o ensino fundamental. Os melhores coeficientes de correlação foram alcançados entre os domínios de fadiga física, geral e total e o questionário de Qualidade de Vida da Organização Mundial da Saúde-BREF e entre os domínios totais e fadiga mental e o Índice de Capacidade para o Trabalho. Correlações fortes também foram alcançadas entre a fadiga geral e total e o escore total do Questionário de Qualidade de Vida Respiratória do Hospital São Jorge.

**Conclusões:**

A silicose e a carga de trabalho física têm influência negativa na qualidade de vida respiratória e na percepção de fadiga dos trabalhadores de mineração.

## INTRODUCTION

Fatigue is one of the most common symptoms in lung diseases such as silicosis, cancer, and chronic obstructive pulmonary disease (COPD), especially as the disease progresses.^1^ Quality of life (QoL) and work capacity are also important indicators when assessing the impact of lung diseases,^2^ as respiratory symptoms, functional impairment, and smoking can negatively affect them.^3^

It should be noted that, in addition to health problems resulting from exposure to silica, artisanal and small-scale miners (ASM) also face unfavorable working conditions, together with increased productivity demands and the need to work in remote locations, often far from family members and without access to support services.^4^ Although lung function is within acceptable values in patients with silicosis, many chronic lung diseases are linked to psychological disorders such as anxiety and depression, with a negative impact on QoL due to the permanent and progressive aspect of these diseases.^5^

Environmental conditions intrinsic to ASM, such as lack of natural light, uncomfortable workspace, particle pollution, low oxygen levels, gas contamination, changes in rock mass structure, noise and vibration of machines and equipment used for extraction and haulage, can negatively impact the psychomotor and mental states of workers.^6^ These conditions contribute to accidents and reduced caution at work^6^ and expose the true impact that disabilities, illnesses, or dysfunctions can have on the affected workers, their families, and the community.

This issue is especially concerning when working in unhealthy environments, such as mining, and should be better understood^7^ to help improve QoL and work capacity and to prolong life expectancy.^8^ Reducing fatigue is also crucial to preserve work capacity and keep workers safe. The objective of this study was to assess whether fatigue influences QoL and work capacity in miners with silicosis.

## METHODS

This was a cross-sectional study with miners from the Northern Region of the state of Rio Grande do Sul, Brazil. Twenty-seven miners diagnosed with silicosis were included in the study. Data were collected during the second half of 2017 and first half of 2018 using the Brazilian version of the following instruments: Questionnaire of Sociodemographic, Lifestyle, and Work and Health Aspects (QSETS),^9^ Yoshitake Fatigue Questionnaire (YFQ),^10^ Work Ability Index (WAl),^11^ St. George’s Respiratory Questionnaire (SGRQ),^12,13^ and the World Health Organization Quality of Life – BREF (WHOQOL-BREF).^14^

All study procedures involving humans were performed according to the ethical standards of the institutional and/or national research ethics committee. Statistical analyses were performed using SPSS® version 22.0 (Chicago, IL, USA). A p-value of < 0.05 was considered statistically significant. Data were expressed as frequency and percentage for categorical variables and as mean ± SD or median and interquartile range for numeric variables. Factors associated with the SGRQ^ and WHOQOL-BREF were evaluated using Spearman’s correlation coefficient. Linear regression analyses were performed considering the relationship between the physical, mental, overall, and total fatigue domains and the SGRQ and WHOQOL-BREF.

## RESULTS

Mean participant age was 52.6 (± 7.2) years, and 81% were married or lived with a partner. All participants were men, and most of them (70.4%) did not finish elementary school. Eleven participants were smokers or former smokers, with a mean time of current/past smoking of 11.6 years. Workers whose first job was in mining accounted for 60% of the sample, with a mean age at first job of 13.8 (± 3.3) years. Mean time working as a miner was 28.7 (± 10.5) years, and the mean WAI score was 35 (± 6.3) points (Table 1).

**Table 1:** Sociodemographic, occupational, and health information of miners with silicosis (n = 27)

Characteristics	Frequency (%)	Mean	SD	Minimum	Median	Maximum
Demographic data						
Age, years	-	52.6	7.2	35.0	54.0	64.0
Marital status, married	22 (81.5)	-	-	-	-	-
Children	-	0.9	0.3	1.0	1.0	4.0
Number of people living in the house	-	2.1	1.2	0.0	2.0	5.0
Height, cm	-	1.7	0.6	1.6	1.7	1.8
Weight, kg	-	69.4	9.3	58.0	70.0	96.0
BMI	-	23.7	2.8	20.4	23.1	33.6
Did not finish elementary school	19 (70.4)	-	-	-	-	-
History of smoking, pack-year	-	11.5	21.9	0.0	0.0	80.0
Alcohol consumption, yes	22 (81.5)	-	-	-	-	-
Age at first job, years	-	13.8	3.3	8.0	14.0	21.0
First job was in mining	14 (51.9)	-	-	-	-	-
Years working as a miner	-	28.7	10.5	11.0	27.0	42.0
Daily working hours	-	7.6	0.1	6.0	8.0	8.0
WAI	-	35.2	6.3	18.0	37.0	44.0
Respiratory diseases	-	-	-	-	-	-
Sinusitis	6 (22.2)	-	-	-	-	-
Rhinitis	4 (14.8)	-	-	-	-	-
COPD	4 (14.8)	-	-	-	-	-
Tuberculosis	2 (7.4)	-	-	-	-	-
Signs and symptoms						
Cough	20 (74.1)	-	-	-	-	-
Sputum	17 (63.0)	-	-	-	-	-
Wheezing	9 (33.3)	-	-	-	-	-
Dyspnea	16 (59.3)	-	-	-	-	-
Chest pain	6 (22.2)	-	-	-	-	-
Weight loss	7 (25.9)	-	-	-	-	-
Loss of appetite	5 (18.5)	-	-	-	-	-
Fever	2 (7.4)	-	-	-	-	-
Epistaxis	2 (7.4)	-	-	-	-	-
Health and QoL						
SGRQ						
Symptoms	-	26.5	16.0	2.0	21.0	68.0
Impact	-	22.2	13.8	0.0	19.8	50.9
Activity	-	19.6	12.8	0.0	23.4	42.9
Total	-	22.8	11.8	4.1	19.6	47.9
WHOQOL-BREF						
Physical health	-	70.6	13.4	42.9	71.4	100.0
Psychological health	-	70.5	12.3	41.7	66.7	100.0
Social relationships	-	71.9	15.0	33.3	75.0	100.0
Environment	-	61.9	9.8	40.6	62.5	87.5
Total	-	91.7	8.7	75.0	91.0	100.0
Fatigue						
Physical*	-	17.6	7.7	10.0	15.0	36.0
Mental†	-	19.1	7.3	10.0	17.0	37.0
Overall‡	-	20.3	7.8	10.0	21.0	34.0
Total	-	57.1	20.8	30.0	48.0	91.0

* Sleepiness and lack of will to work.

† Difficulty concentrating.

‡ Perception of physical fatigue.

BMI = body mass index; COPD = chronic obstructive pulmonary disease; QoL = quality of life; SGRQ = St. George’s Respiratory Questionnaire; WAI = Work Ability Index; WHOQOL-BREF = World Health Organization Quality of Life – BREF.

In addition to silicosis, workers reported other respiratory diseases, such as sinusitis (22.2%), rhinitis (14.8%), COPD (14.8%), and tuberculosis (7.4%). They also described signs and symptoms of cough (74.1%), sputum (63%), wheezing (33.3%), dyspnea (59.3%), chest pain (22.2%), weight loss (25.9%), loss of appetite (18.5%), fever (7.4%), and epistaxis (7.4%) (Table 1).

In the SGRQ, participants scored 26.6 points in the symptoms domain, 22.3 points in the impact domain, and 19.7 points in the activity domain, with a total score of 22.8. As for the domains of the WHOQOL-BREF, participants scored 70.6 points in physical health, 70.5 in psychological health, 71.9 in social relationships, and 61.9 in environment, with a total score of 91.7. Each domain of the YFQ allows a maximum score of 50 points. Participants scored 19.7, 19.1, and 20.4 points in the physical, mental, and general fatigue domains, respectively, with a total score of 57.2 points (Table l).

The correlation coefficients between demographic data and the WHOQOL-Bref, and SGRQ in relation to the YFQ are shown in Table 2.

**Table 2:** Correlation of selected variables, SGRQ domains, and WHOQOL-Bref domains with the YFQ (n = 27)

Characteristics	Physical fatigue	Mental fatigue	Overall fatigue	Total fatigue
Age	−0.286*	0.226*	−0.144*	−0.217*
History of smoking, pack-year	0.176*	0.223*	0.355†	0.273*
Years working as a miner	−0.466†	−0.215*	−0.143*	−0.193∥
Daily working hours	0.335†	0.183*	0.326†	0.344†
Work capacity	−0.481†	−0.608†	−0.508†	−0.560†
SGRQ				
Symptoms	0.392†	0.228*	0.505†	0.458†
Impact	0.381†	0.132*	0.385†	0.417†
Activity	0.292*	0.283*	0.378†	0.398†
Total	0.430†	0.323*	0.525†	0.539†
WHOQOL-BREF				
Physical health	−0.544†	−0.509†	−0.709†	−0.673†
Psychological health	−0.564†	−0.336†	−0.514†	−0.509†
Social relationships	−0.263*	−0.426†	−0.464†	−0.475†
Environment	−0.567†	−0.529†	−0.512†	−0.525†
Total	−0.526†	−0.405†	−0.557†	−0.544†

Spearman correlation, p-value:

* > 0.10.

† 0.05 < p > 0.10.

^‡^p < 0.05.

^§^p < 0.001.

∥ p > 0.10.

SGRQ = St. George’s Respiratory Questionnaire; WHOQOL-BREF = World Health Organization Quality of Life Questionnaire – BREF; YFQ = Yoshitake Fatigue Index.

Stronger negative correlations were found between overall and total fatigue and the physical health domain of the WHOQOL-BREF (r = − 0.709, p < 0.001; r = −0.673, p < 0.001); mental fatigue and the WAI (r = −0.608, p < 0.001); and physical fatigue and the physical health domain of the WHOQOL-BREF (r = −0.544, p < 0.001).

Strong correlations were also observed between overall and total fatigue and the total SGRQscore (r = 0.525, p < 0.001; 0.539, p < 0.001) (Table 2).

Linear regression analysis showed a significant negative association between physical fatigue and the WAI and physical fatigue and the total WHOQOL-BREF score (adjusted R^2^ −0.234, p < 0.05; and adjusted R^2^ −0.285, p < 0.05); and between mental fatigue and the WAI and mental fatigue and the total WHOQOL-BREF score (adjusted R^2^ −0.248, p < 0.05; and adjusted R^2^ −0.218, p < 0.05) (Table 3).

**Table 3:** Correlation between YFQ domains and the WAI, total SGRQ score, and total WHOQOL-BREF score

Characteristics	Physical fatigue		Mental fatigue		Overall fatigue		Total fatigue	
	Adjusted R^2^	P- value	Adjusted R^2^	P- value	Adjusted R^2^	P- value	Adjusted R^2^	P- value
WAI	−0.234	**0.006**	−0.248	**0.005**	−0.283	**0.003**	−0.314	**0.001**
SGRQ – total	0.113	**0.048**	0.009	0.386	0.223	**0.008**	0.119	**0.043**
WHOQOL-BREF – total	−0.285	**0.002**	−0.218	**0.008**	−0.302	**0.002**	−0.330	**0.001**

Bold type denotes statistical significance.

Results of linear regression analysis: SGRQ – total = total St. George’s Respiratory Questionnaire score; WAI = Work Ability Index; WHOQOL-Bref – total = total World

Health Organization Quality of Life Questionnaire-BREF score; YFQ = Yoshitake Fatigue Questionnaire.

A significant negative association was also found between overall fatigue and the WAI, overall fatigue and the total SGRQ score, and overall fatigue and the total WHOQOL-BREF score (adjusted R^2^ −0.283, p < 0.05; adjusted R^2^ 0.223, p < 0.05; and adjusted R^2^ −0.302, p < 0.05); total fatigue and the WAI, total fatigue and the total SGRQ score, and total fatigue and the total WHOQOL-BREF score (adjusted R^2^ -0.314, p < 0.05; adjusted R^2^ 0.119; p < 0.05; and adjusted R^2^ −0.330, p < 0.05) (Table 3).

## DISCUSSION

Mean participant age in this study was higher than that found in another study conducted with miners diagnosed with silicosis.^15^ However, it should be noted that silicosis is a progressive disease that, in a short period of time (10 to 20 years^16^), can significantly impair work capacity affecting QoL and increasing fatigue, including in activities of daily living. Participants started working very early, and most of them reported that their first job was in mining. They also had difficulties staying in school, which can be considered a risk factor for reduced caution at work and increased risk of developing occupational diseases due to inadequate use of protective equipment.^17^ Alcohol consumption and smoking can also be considered risk factors for silicosis, as they increase and accelerate lung damage.^18,19^

SGRQ scores were lower in this study than in those by Gonzalez et al.^2^ and Warren,^18^ which also included miners with lung disease and also considered the same criteria: symptoms (26.56 vs. 34.99 and 50.63, respectively); activity (19.65 vs. 49.14 and 48.26, respectively); impact (22.25 vs. 30.84 and 26.98, respectively); and total (22.82 vs. 37.08 and 37.08, respectively). In our study, the QoL of workers with silicosis, assessed by the WHOQOL-BREF, reached more than 60 points – which can be considered high, especially when considering working conditions. In addition, QoL was higher in the physical and psychological domains, even when compared with scores from a study^20^ evaluating miners without silicosis: 70.63 ± 13.41 vs. 70.51 ± 11.13 in the physical domain and 70.52 ± 12.30 vs. 68.73 ± 11.39 in the psychological domain. However, the environment domain had one of the lowest scores in the questionnaire. This domain includes issues related to safety and protection in the physical environment, family environment, financial aspects, health care and social interaction, access to new information and skills development, access to leisure and recreational activities, and the environment. This shows that, although QoL was considered high by the WHOQOL-BREF, workers’ perception of the workplace and daily life influences and reduces QoL scores.^21^

Participants also showed low self-reported fatigue, especially in relation to physical and mental fatigue, compared with participants from other studies.^1,22^ However, the fatigue domains reached a strong negative association with some of the WHOQOL-BREF domains and the WAI. In addition, the overall and total fatigue domains showed a strong association with the total SGRQ score. Considering that the higher the SGRQ score, the worse the levels of QoL related to breathing and fatigue, our results are consistent with other studies.^23,24^ Furthermore, silicosis can cause disability and lead to time off work and early retirement; it can also compromise QoL, especially in workers with advanced stages of the disease.^25,26^

Our study has some limitations. This was the first study to use the YFQ to assess fatigue in miners, which may hinder the comparison of results with other studies. Second, we included a very small sample size, and participants did not undergo clinical evaluation, such as spirometry, chest X-ray, or computed tomography. Third, mining is characterized as heavy manual work in the study region, which may have influenced workers’ responses to questions related to fatigue and respiratory QoL, as they may vary according to individual, social, and cultural factors. Thus, the results should be interpreted with caution, as participants are still actively working, whereas other studies included retired workers or workers on leave.

## CONCLUSIONS

Silicosis and physical overload can negatively influence respiratory QoL and the perception of fatigue in miners. This relationship was confirmed by the negative correlation between the WHOQOL-BREF, WAY, and YFQ domains. However, our results showed higher QoL scores and lower fatigue, even among participants diagnosed with silicosis.
